# Consensus Statements on Managing Aesthetic Needs in Prescription Medication‐Driven Weight Loss Patients: An International, Multidisciplinary Delphi Study

**DOI:** 10.1111/jocd.70094

**Published:** 2025-03-26

**Authors:** Andreas Nikolis, Kaitlyn M. Enright, Sabrina G. Fabi, Michael Somenek, Hugues Cartier, Luiz Avelar, Johnny Franco, Alessandra Haddad, Maria Angelo‐Khattar, Jeff Huang, Tyler Safran, Inna Prygova, Steven Dayan

**Affiliations:** ^1^ Clinical Research Unit Erevna Innovations Inc Westmount Quebec Canada; ^2^ Division of Plastic and Reconstructive Surgery McGill University Montréal Québec Canada; ^3^ Cosmetic Laser Dermatology, San Diego University of California—San Diego San Diego California USA; ^4^ Somenek+Pittman MD Advanced Plastic Surgery Washington DC USA; ^5^ Saint‐Jean Dermatology Center Arras France; ^6^ DOMANI Clinic Belo Horizonte Brazil; ^7^ Austin Plastic Surgeon Austin Texas USA; ^8^ Department of Plastic Surgery Federal University of São Paulo UNIFESP Sao Paulo Brazil; ^9^ AHaddad Medicine and Surgery Clinic Sao Paulo Brazil; ^10^ Altaderma Clinic Dubai UAE; ^11^ American Academy of Anti‐Aging Medicine Boca Raton Florida USA; ^12^ L'excellence Clinica Taipei Taiwan; ^13^ Galderma Uppsala Sweden; ^14^ Department of Otolaryngology University of Illinois Chicago Illinois USA

**Keywords:** anti‐obesity drugs, glucose‐lowering drugs, obesity, ozempic‐face, type 2 diabetes

## Abstract

**Background:**

To handle the increasing influx of prescription medication‐driven weight loss (mdWL) patients in aesthetic practices, clinicians must be aligned on identifying discerning factors and strategies for managing this unique patient population.

**Objectives:**

(1) Define the mdWL patient; (2) describe the mdWL patient's aesthetic expectations; (3) determine the most relevant methods of assessing mdWL patients in clinical practice; (4) determine the effects of mdWL on specific facial tissue layers; (5) identify important treatment considerations for the mdWL patient; and (6) identify the temporal sequencing of non‐surgical options in the mdWL patient.

**Methods:**

Preparatory research included patient interviews, market research, and a systematic literature review. Following this, an international, multidisciplinary three‐round Delphi study was conducted to collect information on practice setting, physician and patient demographics, and previous experience, and for panelists to vote on consensus statements regarding managing mdWL patients in aesthetics.

**Results:**

mdWL is best defined by the percent of BMI lost within ≤ 6 months. Three‐dimensional volumetric analysis is an effective quantitative assessment, while photo‐numeric scales and patient‐reported outcome measures are relevant qualitative measures. Tissue layers most affected by mdWL include the skin and superficial and deep fat pads. A major concern for aesthetic mdWL patients seeking aesthetic treatments is the fear of appearing to have gained weight following treatments, while for physicians it is ensuring their mdWL patients look healthy and natural. The key selection and critical timing of aesthetic treatments throughout the mdWL journey are described.

**Conclusions:**

The first global consensus‐based guidelines for understanding and managing the aesthetic needs of mdWL patients are presented.

## 1 Introduction

Worldwide, the prevalence of obese and overweight adults is increasing and is now considered a global epidemic [1]. Relatedly, the number of patients accessing prescription weight loss medications (e.g., glucagon‐like peptide‐1 [GLP‐1] agonists such as liraglutide, dulaglutide, semaglutide, and tirzepatide) continues to rise [2, 3]. The increase in prescription medication‐driven weight loss (mdWL) patients is thought to be associated with off‐label prescribing of pharmacological medication for the management of type‐2 diabetes, for weight loss in people without the disease, and is fueled by social media interest [4, 5, 6]. Interestingly, a cross‐sectional study using data from medical and insurance claims and electronic health records found that obesity prevalence in the US decreased in 2023 for the first time in over a decade [7].

Given the facial changes associated with mdWL (e.g., increased skin laxity, sagginess, pronounced wrinkles and folds) [3, 8, 9, 10], many patients seek aesthetic improvements [9, 11]. To handle the increasing influx of mdWL patients in aesthetic practices, clinicians must be educated on identifying discerning factors and managing this unique patient population [12]. This becomes even more relevant given recent data from the American Med Spa Association (AMSPA), which indicates that half of all med spas now offer a weight loss solution (e.g., GLP‐1 medications, IV drips, hormone replacement) [13]. Moreover, an April 2024 survey of 722 medical aesthetic clinics found that 72% offered GLP‐1 medications for weight loss [13].

Given the surge in popularity of mdWL medications, high‐quality research such as randomized controlled trials are lacking. Therefore, for such a heterogeneous topic (i.e., the aesthetic needs of mdWL patients), the application of methods aiming to increase the homogeneity of clinical guidelines is useful and appropriate. Consensus methods are often chosen when evidence is absent, inadequate/limited, contradictory, or emerging in existing research literature. When no robust evidence is available, there is a need for collective judgment to increase the reliability and validity of guidelines for clinical decision‐making, and such approaches may be formulated based on expert consensus only [14]. For this reason, a Delphi study was conducted to reach a consensus among industry experts. Using this approach, the panelists discussed different clinical scenarios and elaborated statements based on the published literature and their clinical experience. The objectives of this consensus meeting were to:

1. Define the mdWL patient.
2. Determine the most relevant methods of assessing mdWL patients in research and clinical practice.
3. Determine if mdWL impacts specific facial tissue layers.
4. Identify important treatment considerations for the mdWL patient.
5. Identify the temporal sequencing of non‐surgical options in the mdWL patient.
6. Describe the mdWL patient's aesthetic expectations.

## 2 Materials and Methods

**FIGURE 1:**
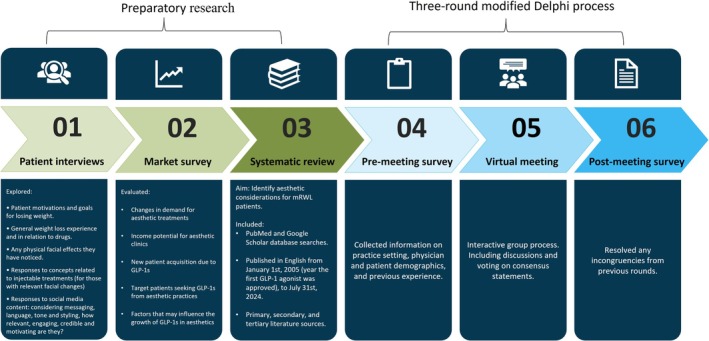
An overview of each step conducted throughout the consensus process.

The consensus project comprised six steps: (1) interviews of mdWL patients to gather information from the patients' perspective; (2) a market survey conducted to evaluate factors influencing the growth of mdWL patients in aesthetics; (3) a systematic literature review to identify aesthetic considerations for mdWL patients; and (4–6) three rounds of an online modified Delphi consensus process to develop and validate the selected statements. An overview of each consensus step is displayed in Figure 1.

### 2.1 Registration

Prospective registration of the planned protocol for a consensus exercise is recommended as best practice for increased transparency. In addition, public registration of intended research can help to avoid duplication and prevent research waste [14]. Therefore, the study protocol was registered on the Open Science Framework (unique identifier: https://doi.org/10.17605/OSF.IO/35HE2).

### 2.2 Steering Committee and Selection of Panelists

A steering committee oversaw the project's organizational initiatives and directed the consensus exercise. The committee consisted of key stakeholders (e.g., representatives from the endorsing organization), senior leadership, and project managers with expertise in consensus methods. Responsibilities of the steering committee included topic selection, establishing the budget, determining resource allocation, project timelines, selection of panelists, setting expectations and milestones, study monitoring, defining project outcomes and key audiences (i.e., aesthetic clinicians located across the globe), developing the meeting agenda and materials, advanced preparation of statements and questions, and summarizing existing scientific evidence to present to the panelists.

The “closeness continuum” developed by Needham and de Loë (1990), was applied as a framework for including experts who had the subjective (experiential knowledge or real‐life experiences), mandated (those with professional, legal, and/or ethical responsibility), and objective (those who study the topic [e.g., specialized clinicians and researchers]) expertise and experience to make a positive contribution to the topic of interest [15]. Panelists were recruited via electronic invitations sent from the lead author. The number of panelists (*N* = 10) was selected to ensure diversity among the panel guests in terms of ethnicity, gender, and multidisciplinary expertise. The geographical locations of the panelists' practices were considered to ensure representation of countries where medical weight loss medications were accessible and in high demand. Recruitment was restricted to the invited panelists (i.e., invitees could not recruit more people [snowballing]). An overview of the roles of each participant is listed in Supporting Information Table S1.

### 2.3 Preparatory Research

Prior to the consensus exercise, patient interviews (conducted by MAC Research), a market survey (obtained by Medical Insight Inc.), and a systematic literature review were performed to generate items and other meeting materials and provide panelists with a summary of existing scientific evidence. The preparatory research provided to panelists was obtained from multiple sources (i.e., bibliographic research, patient interviews, survey responses), which may have offered additional relevant information compared to summaries from a single source. A description of the methodology and results of the systematic review is described below. A summary of the patient interviews and market survey is also presented, although a detailed description is reported elsewhere [13, 16].

#### 2.3.1 Patient Interviews

Twelve (*N* = 12) 1‐h online interviews were conducted among mdWL patients in the USA [17]. Respondents were recruited from a market research database. All patients had lost weight using prescribed weight loss medications (on and off‐label indications). The total amount of body weight lost ranged from 5% to > 20%. Patients with different ethnic backgrounds were recruited (i.e., Caucasian, African American, Asian, Hispanic) with a mean age of 40.2 (range: 25–55 years). The sample included eight females (66.66%) and four males (33.33%). Half (*n* = 6) of the patients had previous experience undergoing aesthetic treatments, while the other half had not but were open to the idea. Interview questions were formulated to explore the following concepts:

- Patient motivations and goals for losing weight.
- General weight loss experience and in relation to prescription medications.
- Any physical facial effects they have noticed.
- Responses to concepts related to injectable treatments (for those with relevant facial changes).
- Responses to social media content: considering messaging, language, tone, and styling, how relevant, engaging, credible, and motivating are they?

#### 2.3.2 Market Survey

A market survey report was compiled from public and proprietary sources [13]. Information was cross‐checked against other data using forecasting models and synthesized into qualitative and quantitative analyses and projections. Public sources utilized for this report includedarticles in trade publications, medical journals, and regulatory documents; articles in consumer magazines and newspapers; company news releases, website information, patent documents, regulatory data, marketing materials, and financial filings; and information from trade associations. Proprietary sources utilized includedan internal database of industry and product information; industry analyst reports; and exclusive interviews with company executives, analysts, researchers, sales representatives, physicians, patients, consultants, and other industry experts [13, 16].

#### 2.3.3 Systematic Literature Review

The Population, Intervention, Comparison (if relevant), Outcome, and Timing of measurement (PICOT) approach was used for framing the research question, as this has been independently associated with better overall reporting quality [18]. The population (P) included mdWL patients, the intervention (I) included prescription weight loss medications, the use of comparators was not applicable, the outcome of interest was aesthetic facial changes, and the timing of the outcomes was within 6 months of weight loss. The research question of the systematic literature review was “What are the facial aesthetic needs of prescription medication‐driven weight loss patients?”

For performing the systematic review, an electronic search of the PubMed database (https://pubmed.ncbi.nlm.nih.gov/) was conducted on August 1st, 2024 for articles published in English from January 1st, 2005 (the year the first GLP‐1 agonist was approved for clinical use) [13], to July 31st, 2024. An additional search of Google Scholar was done on August 15th, 2024, and the first 250 titles were screened. Filters applied included: Full text, Case Reports, Clinical Study, Clinical Trial, Clinical Trial Phase I, Clinical Trial Phase II, Clinical Trial Phase III, Clinical Trial Phase IV, Comparative Study, Controlled Clinical Trial, English Abstract, Equivalence Trial, Evaluation Study, Guideline, Meta‐Analysis, Multicenter Study, Observational Study, Randomized Controlled Trial, Review, Systematic Review, Validation Study, English, Humans, Adult: 19+ years, from 2005/1/1–2024/7/31. The research question was translated into keywords for the search. Synonyms/alternate terms and different spellings were considered. Keywords were also combined with Boolean operators (i.e., the words “AND”, “OR” and “NOT”). Keywords included: aesthetics, cosmetics, plastic surgery, hyaluronic acid, poly‐l‐lactic acid, calcium hydroxylapatite, botulinum toxin, dermatology, bariatric, facial, and weight loss. Hierarchical vocabulary (i.e., Medical Subject Heading [MeSH]), which indexes articles that use different terminology for identical ideas, was used to increase the yield of articles via “Automatic term mapping” and “automatic term explosion”. These features match keywords with MeSH transcription table headings and then explode into various subheadings [19].

Primary literature sources included publications (e.g., clinical trials, case reports/series) in peer‐reviewed journals. Secondary sources included systematic reviews or meta‐analyses where material derived from primary source literature was inferred and evaluated. Tertiary literature consisted of a collection that compiled information from primary or secondary literature (e.g., reference lists, personal knowledge of landmark studies, incidental discovery, browsing through the link entitled “Related Articles” [a PubMed feature that searches for similar citations using an intricate algorithm that scans titles, abstracts and MeSH terms]). Trials registries and gray literature sources were not searched, nor were authors contacted due to resource limitations. The evidence retrieved was manually reviewed by the steering committee for inclusion in the panelists' pre‐meeting information pack. Zotero was used to manage references [20]. Copies of the selected references were made available to the panelists before the consensus meeting. Review of the reading material was required prior to the group meeting (step 5).

### 2.4 Assessing Consensus

An international working group of experts from Asia, Europe, and North & South America was formed to reach a consensus on clinical guidance, nomenclature, and other approaches related to treating mdWL in aesthetics. Defining who an expert is, and thus who should participate in a consensus, is crucial to the success of any Delphi exercise. However, the concept of “expert” is contested [15]. We implemented a framework that combined traditional and evolving definitions of expertise and experience to select an inclusive population of independent and heterogeneous specialists with the necessary knowledge to reach a consensus on key topics related to treating mdWL patients in aesthetics. Then, an electronic modified Delphi (e‐Delphi) method was used to formulate consensus statements using three rounds of surveys/polls. Three characteristics defined the e‐Delphi consensus method used in this study: anonymity, iteration (over multiple rounds of voting), and controlled feedback. A structured and systematic e‐Delphi method was used to develop consensus statements, instead of more informal techniques that can lack methodological rigor (e.g., focus groups) [14]. For example, in an unstructured group meeting, there is the risk of a single individual dominating the discussion, and decisions may be portrayed as unanimous when in actuality, there is dissent within the group. With a structured consensus meeting, idiosyncrasies are transparently reported. First, a pre‐meeting survey collected information on practice setting, physician and patient demographics, and previous experience. Furthermore, the first round was used as a “brainstorming” round before voting to draft statements using the amalgamation of ideas presented by the panel members. Then during the meeting (September 2024), the results of the pre‐meeting survey were presented, and discussion was prompted by evidence from the literature, or lack thereof. Additionally, advisors responded to polling questions regarding managing mdWL patients in aesthetics. A moderator ensured each participant was given the opportunity to speak and vote. Panelists were provided opportunities to suggest rewordings to the statements throughout the Delphi process. The steering committee ultimately determined whether suggestions for a reworded item were retained for the next survey round. Consensus was considered met (high agreement) when ≥ 7/10 of panel members agreed, consensus was unmet (low agreement) when < 6/10 to ≥ 4/10 of panel members agreed, and no consensus was reached when < 3/10 of panel members agreed. A post‐meeting survey resolved incongruities. Surveys/polls and the meeting took place in English. Individual responses to Delphi rounds were deidentified at the source level by the platforms used (SurveyMonkey Inc. [San Mateo, California, USA]; Zoom Video Communications Inc. [Version 6.2.6; San Jose, California, USA]). A mixed methods approach was used for data analysis, where qualitative methods were used when comments, suggestions, perceptions, cases, and experiences were collected, and quantitative methods were used when responses/votes were aggregated and summarized. The present manuscript was prepared following guidelines for reporting consensus methods used in biomedical research (i.e., ACcurate COnsensus Reporting Document [ACCORD]; refer to the checklist in Supporting Information Table S2).

## 3 Results

### 3.1 Systematic Review

**FIGURE 2:**
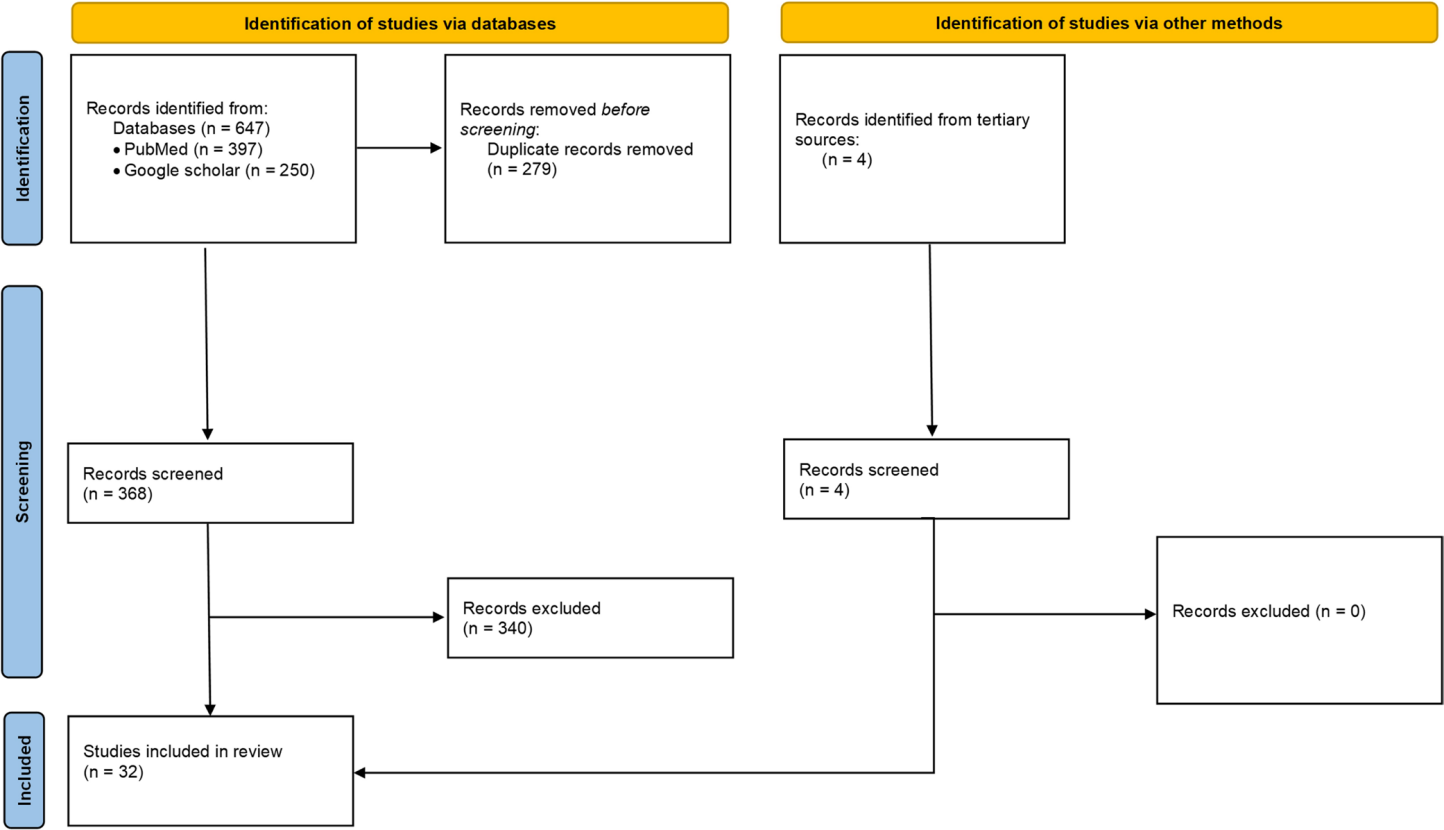
Preferred Reporting Items for Systematic reviews and Meta‐Analyses (PRISMA) flow diagram for new systematic reviews which included searches of databases, registers and other sources. The PRISMA flow diagram template is distributed in accordance with the terms of the Creative Commons Attribution (CC BY 4.0) license. To view a copy of this license, visit https://creativecommons.org/licenses/by/4.0/.

A flow diagram (Figure 2) depicts the flow of information through the different phases of a systematic review and maps out the number of records retrieved, screened, included, and excluded.

### 3.2 Member Demographics and Clinical Populations Treated

**FIGURE 3:**
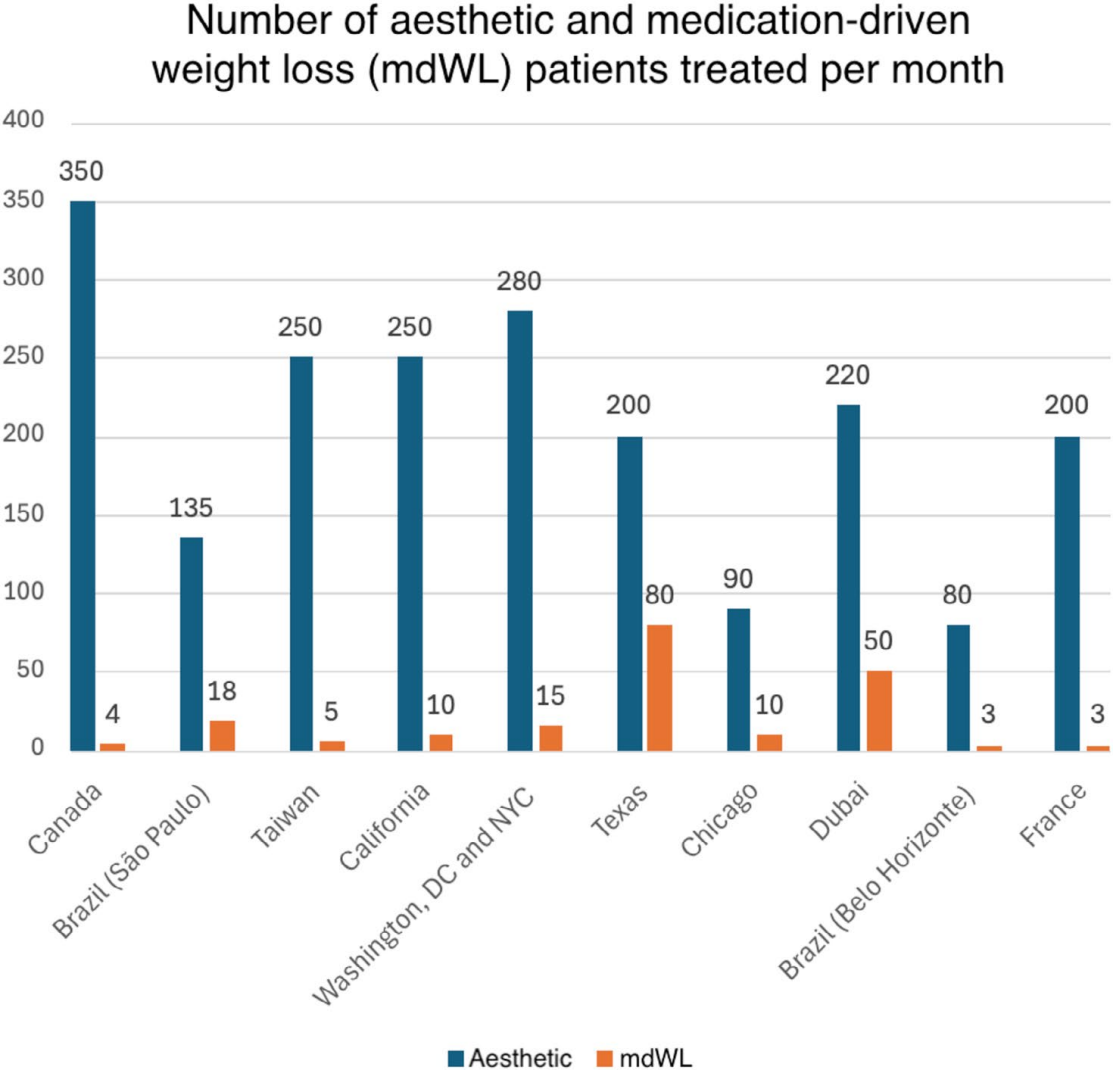
The number of aesthetic and prescription medication‐driven weight loss patients treated per month by each clinician‐panelist. Panelists may treat patients at multiple clinic locations.

Clinician members had an average of 20.3 (SD: 6.7; Range: 11–30) years of experience and included surgeons (*n* = 6), dermatologists (*n* = 3), and aesthetic physicians (*n* = 1). The average (Mean, [SD]) number of aesthetic and mdWL patients treated per month was 205 (80.45) and 13.1 (14.05; Figure 3), respectively, making the average percentage of mdWL patients in these aesthetic practices 6.4% of the clinic population. The ratio of female to male patients was 8.5–1.5. The average age of mdWL patients was 43.00 (5.03) years. The ethnic distributions (%) of aesthetic and mdWL patients are displayed in Table 1.

**TABLE 1:** The ethnic distributions (%) of aesthetic and prescription medication‐driven rapid weight loss patients treated by the panelists.

Ethnicity	Demographic distributions (%) of aesthetic patients	Demographic distributions (%) of RWL patients
Aboriginal	0.30 (0.90)	Nil.
Middle Eastern	13.89 (22.46)	13.00 (28.04)
Black	5.30 (5.69)	2.30 (3.26)
Asian	14.00 (27.51)	11.50 (29.67)
Filipino	0.78 (0.92)	0.60 (1.50)
Latin	23.70 (25.71)	16.50 (27.02)
White	44.50 (28.03)	56.10 (37.79)

### 3.3 Pre‐ and Post‐Meeting Surveys

For both the pre‐and post‐meeting surveys, there was a 100% response rate (*N* = 10), and no questions were skipped. Survey questions and answer summaries are presented in Tables 2 and 3. Raw data and responses to open‐ended questions are presented in Supporting Information Tables S3, S4.

**TABLE 2:** Pre‐meeting survey questions and answer summaries.

N^o^	What is your specialty? (*N* = 10)		
Q1	Plastic surgery	Dermatology	Aesthetic medicine
	60% (*n* = 6)	30% (*n* = 3)	10% (*n* = 1)
Q2	How many years have you been practicing aesthetics? (*N* = 10)		
	20.20 years (SD: 6.95)		
Q3	How many aesthetic patients do you treat per month? (*N* = 10)		
	205.50 patients (SD: 84.80)		
Q4	What proportion (%) of you aesthetic patients are female? (*N* = 10)		
	84.5%		
Q5	What proportion (%) of you aesthetic patients are male? (*N* = 10)		
	15.5%		
Q6	What is the mean age of your aesthetic patients (approximate)? (*N* = 10)		
	46.5 years (SD: 4.06)		
Q7	How many rapid weight loss (RWL) patients do you treat for aesthetic indications per month? (*N* = 10)		
	19.80 patients (SD: 25.35)		
Q8	What proportion (%) of you RWL patients are female? (*N* = 10)		
	89.4%		
Q9	What proportion (%) of you RWL patients are male? (*N* = 10)		
	10.6%		
Q10	What is the mean age of your RWL patients (approximate)? (*N* = 10)		
	43.00 years (SD: 5.33)		
Q11	What parameters are important to consider for the development of an aesthetic treatment algorithm for RWL patients (1 = most important, 10 = least important)? (*N* = 10)		
	Refer to Figure 4 for a summary of responses.		

*Note:* Data represented as a mean [+/− standard deviation (SD)]. Additional survey elements (e.g., responses to opened ended questions) are available in Supporting Information X.

**FIGURE 4:**
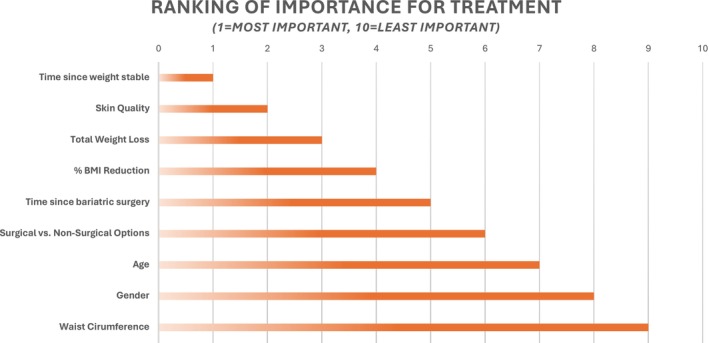
Pre‐Survey Most Important Parameters to Consider for Development of an Aesthetic Treatment Algorithm for MWL.

**TABLE 3:** Post‐meeting survey questions and answer summaries.

Q1. Should the term “Ozempic face” be replaced with Rx‐RWL for the purposes of (1) avoiding brand names and (2) differentiating between bariatric and medication‐induced RWL? (*n* = 10)									
Yes				No					
90% (*n* = 9)				10% (*n* = 1)					
Q2. With regards to body indications, which areas are appropriate to evaluate and treat with poly‐L‐lactic acid (e.g., Sculptra; PLLA‐SCA) and/or Skinboosters in the RWL patient? (*n* = 10)									
Chest and décolle‐tage	Abdomen including flanks	Buttocks	Thighs (anterior and posterior)	Knees		Arms	Hands	Back	Other*
100% (*n* = 10)	60% (*n* = 6)	80% (*n* = 8)	70% (*n* = 7)	70% (*n* = 7)		80% (*n* = 8)	50% (*n* = 5)	20% (*n* = 2)	10% (*n* = 1)
Q3. When would you start treatment with PLLA‐SCA, along the RWL journey, assuming the patient just started weight loss medications? (*n* = 10)									
Concurrently			0–3 months after weight loss			3–6 months after weight loss			
70% (*n* = 7)			10% (*n* = 1)			20% (*n* = 2)			
Q4. What is the degree of impact of RWL on facial muscles? (*n* = 10)									
None			Low			Moderate			
10% (*n* = 1)			70% (*n* = 7)			20% (*n* = 2)			
Q5. Assuming we develop an aesthetic treatment plan for the RWL patient [Rx medical weight loss (e.g., GLP‐1 agonists)], should we stratify recommendations by (please select all that apply): (*n* = 10)									
Gender	Ethnicity	Age	% weight loss	Skin thickness	% BMI change over time	Face functionality		Gender, ethnicity and age	
60% (*n* = 6)	60% (*n* = 6)	100% (*n* = 10)	10% (*n* = 5)	10% (*n* = 1)	10% (*n* = 1)	10% (*n* = 1)		10% (*n* = 1)	
Q6. Does RWL induced by Rx medications (e.g., GLP‐1 agonists) result in similar facial changes to that of HIV‐induced lipoatrophy? (*n* = 10)									
Yes				No					
40% (*n* = 4)				60% (*n* = 6)					
Q7. Should ultrasound be incorporated in pre/post assessments of the RWL patient? (*n* = 10)									
No		Yes, but only for research purposes		Yes, for both standard of care (i.e., routine clinic use) and research purposes					
10% (*n* = 1)		80% (*n* = 8)		10% (*n* = 1)					
Q8. Would you treat a RWL patient with fat grafting? (*n* = 10)									
Yes				No					
50% (*n* = 5)				50% (*n* = 5)					
Q9. If you answered “YES” to the previous question, when would you start treatment with fat grafting, along the RWL journey? (*n* = 5)									
Concurrently		3–6 months after weight is stable		6–9 months after weight is stable		9–12 months after weight is stable			
20% (*n* = 1)		20% (*n* = 1)		20% (*n* = 1)		40% (*n* = 2)			
Q10. Do you think fat transfers are as effective in RWL patients, as in other aesthetic patients who qualify for such treatments? (*n* = 10)									
Yes				No*					
50% (*n* = 5)				50% (*n* = 5)					
Q11. Should major [significant weight loss over a longer period of time (e.g., 1+ years] versus rapid weight loss [significant weight loss over a shorter period of time (e.g., 0–6 months)] be differentiated clinically? (*n* = 10)									
Yes				No					
80% (*n* = 8)				20% (*n* = 2)					

Case #1: The following questions were answered assuming the patient under consideration was a 65‐year‐old female patient who lost 30 lbs over 6 months:			
Q12. What is the degree of impact of RWL on the skin? (*n* = 10)			
Low	Moderate	Significant	
10% (*n* = 1)	20% (*n* = 2)	70% (*n* = 7)	
Q13. What is the degree of impact of RWL on the superficial fat pads? (*n* = 10)			
Low	Moderate	Significant	
0% (*n* = 0)	50% (*n* = 5)	50% (*n* = 5)	
Q14. What is the degree of impact of RWL on the deep fat pads? (*n* = 10)			
Low	Moderate	Significant	
10% (*n* = 1)	20% (*n* = 2)	70% (*n* = 7)	
Q15. What is the degree of impact of RWL on the ligaments? (*n* = 10)			
None	Low	Moderate	
40% (*n* = 4)	40% (*n* = 4)	20% (*n* = 2)	
Q16. What is the degree of impact of RWL on the bones? (*n* = 10)			
None	Low	Significant	
40% (*n* = 4)	50% (*n* = 5)	10% (*n* = 1)	
Q17. What is the degree of impact of RWL on the muscles? (*n* = 10)			
None	Low	Moderate	
10% (*n* = 1)	60% (*n* = 6)	30% (*n* = 3)	
Case #2: The following questions were answered assuming the patient under consideration was a 45‐year‐old male patient who lost 30 lbs over 6 months:			
Q18. What is the degree of impact of RWL on the skin? (*n* = 10)			
Low	Moderate	Significant	
40% (*n* = 4)	50% (*n* = 5)	10% (*n* = 1)	
Q19. What is the degree of impact of RWL on the superficial fat pads? (*n* = 10)			
Low	Moderate	Significant	
30% (*n* = 3)	60% (*n* = 6)	10% (*n* = 1)	
Q20. What is the degree of impact of RWL on the deep fat pads? (*n* = 10)			
Low	Moderate	Significant	
10% (*n* = 1)	60% (*n* = 6)	30% (*n* = 3)	
Q21. What is the degree of impact of RWL on ligaments? (*n* = 10)			
None	Low	Moderate	
30% (*n* = 3)	50% (*n* = 5)	20% (*n* = 2)	
Q22. What is the degree of impact of RWL on the bones? (*n* = 10)			
None	Low	Moderate	
50% (*n* = 5)	50% (*n* = 5)	0% (*n* = 0)	
Q23. What is the degree of impact of RWL on the muscles? (*n* = 10)			
None	Low	Moderate	
10% (*n* = 1)	70% (*n* = 7)	20% (*n* = 2)	
Case #3: The following questions were answered assuming the patient under consideration was a 65‐year‐old male who lost 30 lbs over 6 months:			
Q24. What is the degree of impact of RWL on the skin? (*n* = 10)			
Low	Moderate	Significant	
0% (*n* = 1)	50% (*n* = 5)	50% (*n* = 5)	
Q25. What is the degree of impact of RWL on the superficial fat pads? (*n* = 10)			
Low	Moderate	Significant	
0% (*n* = 1)	70% (*n* = 7)	30% (*n* = 3)	
Q26. What is the degree of impact of RWL on the deep fat pads? (*n* = 10)			
Low	Moderate	Significant	
0% (*n* = 1)	40% (*n* = 4)	60% (*n* = 6)	
Q27. What is the degree of impact of RWL on ligaments? (*n* = 10)			
None	Low	Moderate	Significant
30% (*n* = 3)	50% (*n* = 5)	10% (*n* = 1)	10% (*n* = 1)
Q28. What is the degree of impact of RWL on the bones? (*n* = 10)			
None	Low	Moderate	Significant
40% (*n* = 4)	50% (*n* = 5)	0% (*n* = 0)	10% (*n* = 1)
Q29. What is the degree of impact of RWL on the muscles? (*n* = 10)			
None	Low	Moderate	
10% (*n* = 1)	50% (*n* = 5)	40% (*n* = 4)	

*Note:* During the consensus meeting, questions were answered assuming the patient under consideration was the “standard” RWL patient, described as being a 45‐year‐old female who lost 30 lbs over 6 months.

Abbreviations: GLP‐1 = glucagon‐like peptide‐1 receptor agonist, PLLA‐SCA = poly‐L‐lactic acid, RWL = rapid weight loss.

^a^ Questions 12–17 were answered for a 65‐year‐old female patient who lost 30 lbs over 6 months.

^b^ Questions 18–23 were answered for a 45‐year‐old male patient who lost 30 lbs over 6 months.

^c^ Questions 24–29 were answered for a 65‐year‐old male who lost 30 lbs over 6 months.

### 3.4 Consensus Meeting: Group Discussion

#### 3.4.1 Defining mdWL

Panelists preferred defining mdWL by percent BMI lost (e.g., > 10% of body weight), rather than total weight (lbs/kg) lost. The time requirement for weight loss to be considered “rapid” was generally considered to be 3–6 months. Although recognized as important demographic elements, advisors did not think that the definition of rapid weight loss (RWL) should be dependent on age or sex/gender.

#### 3.4.2 Patient Expectations

Panelists reported that patients who have not yet begun their aesthetic journey after major RWL (e.g., 20+ lbs/10 + kg) complain most of skin that feels loose or saggy, hollowed‐out cheeks, sunken areas beneath the eyes, and more pronounced lines that run between the nose and mouth. Although being a primary treatment concern to panelists, skin issues (e.g., texture, glow) are rarely mentioned by patients themselves to panelists as major concerns. This may reflect a need for clinicians to educate mdWL patients on the effects of RWL on the skin. Prior to starting aesthetic treatments, most mdWL patients report to panelists that they feel like they look older than their actual age. The main reason some mdWL patients have refused aesthetic treatments is due to a fear of appearing to have gained weight. This fear often outweighs concerns about treatment safety and efficacy, cost, and recovery time.

#### 3.4.3 Effect of mdWL on Tissue Layers

Panelists believed that the skin layers most strongly affected by mdWL are the superficial and deep fat pads. Panelists were 50/50 on which fat pad (superficial or deep) is affected first/most. This was identified as a future research direction with imaging studies (e.g., MRI, ultrasound). The importance of the skin as an endocrine organ, even in postmenopausal women, can be affected by mdWL. The effects on the bones and ligaments were considered low. The effect of mdWL on muscles was recognized as possible but undefined in the literature, keeping in mind that despite loss of muscle volume in general, with mdWL, the shear muscle volume in the face is small.

#### 3.4.4 Methods of Assessment

Panelists believed quantitative methods that should be used pre/post‐treatment for assessing RWL patients aesthetically are three‐dimensional (3D) volumetric changes (imagery), the Pinch and Slide Tests [21], and cutometers (measures elasticity) or other objective skin quality measurement devices. Ultrasound may also be useful for measuring volume loss, although this technology/expertise may not be available in all clinics. Qualitative methods should include the Global Aesthetic Improvement Scale (GAIS), validated scales, and patient‐reported outcome measures (PROMs) including quality of life. One of the major agreements (100%) was the need for a specific scale for managing the aesthetic needs of the mdWL patients.

#### 3.4.5 Pre‐Treatment Considerations

Panelists agreed that, where appropriate, the psychological and emotional factors of mdWL patients need to be considered in the treatment plan. Although this is often assessed by the treating aesthetic physician, other specialties may also be consulted (e.g., nutritionist/dietician, psychologist/psychiatrist, endocrinologist). The importance of nutritional guidance was recognized for mdWL patients, although the absolute need for referral to a nutritionist/dietician was debated and likely would be decided on a case‐by‐case basis.

#### 3.4.6 Treatments and Outcomes

Panelists felt that different aesthetic treatments were appropriate for use during different phases of the mdWL journey. For example, while injectables (e.g., neurotoxins, skinboosters, collagen‐stimulators, fillers), energy‐based devices (e.g., lasers, high‐intensity focused ultrasound, radiofrequency), and topical treatments (e.g., microneedling, dermabrasion, facials, chemical peels) were thought to be suitable throughout the RWL journey, definitive aesthetic surgeries should only be considered at a minimum of 6 months after weight has stabilized. No consensus could be reached regarding recommendations for the use of fat transfer in mdWL patients, given the evolving and yet‐to‐be‐confirmed effects of RWL on fat metabolism, adipocyte‐specific factor stimulation, and the potential for weight regain. Overall, it was agreed that employing a comprehensive, multi‐modal treatment approach is critical for the aesthetic management of mdWL patients.

### 3.5 Consensus Statements

#### 3.5.1 Consensus Met (High Agreement, Where ≥ 7/10 of Panel Members Agreed)

1. “Ozempic face” should be replaced by non‐branded terms such as “mdWL patient”: 9/10 panel members agreed.
2. A major concern for physicians is ensuring their mdWL patients look healthy/natural: 9/10 panel members agreed.
3. A major concern for mdWL patients seeking aesthetic treatments is the fear of appearing to have gained weight: 7/10 panel members agreed.
4. Tissue layers most affected by mdWL include skin, superficial, and deep pads: 7/10 panel members agreed.

#### 3.5.2 Consensus Unmet (Low Agreement, Where < 6/10 to ≥ 4/10 of Panel Members Agreed

5 mdWL is best defined by the percentage of BMI lost within ≤ 6 months: 6/10 panel members agreed.
6 Three‐dimensional volumetric analysis is an effective quantitative assessment for evaluating pre‐/post‐treatment effects in mdWL patients: 6/10 panel members agreed.
7 Photo‐numeric scales and patient‐reported outcome measures are relevant qualitative measures for evaluating pre‐/post‐treatment effects in mdWL patients: 6/10 panel members agreed.

#### 3.5.3 No Consensus (Where < 3/10 of Panel Members Agreed)

8 Fat transfer is typically an appropriate treatment for mdWL patients: 3/10 panel members agreed.

### 3.6 Case Series

**FIGURE 5:**
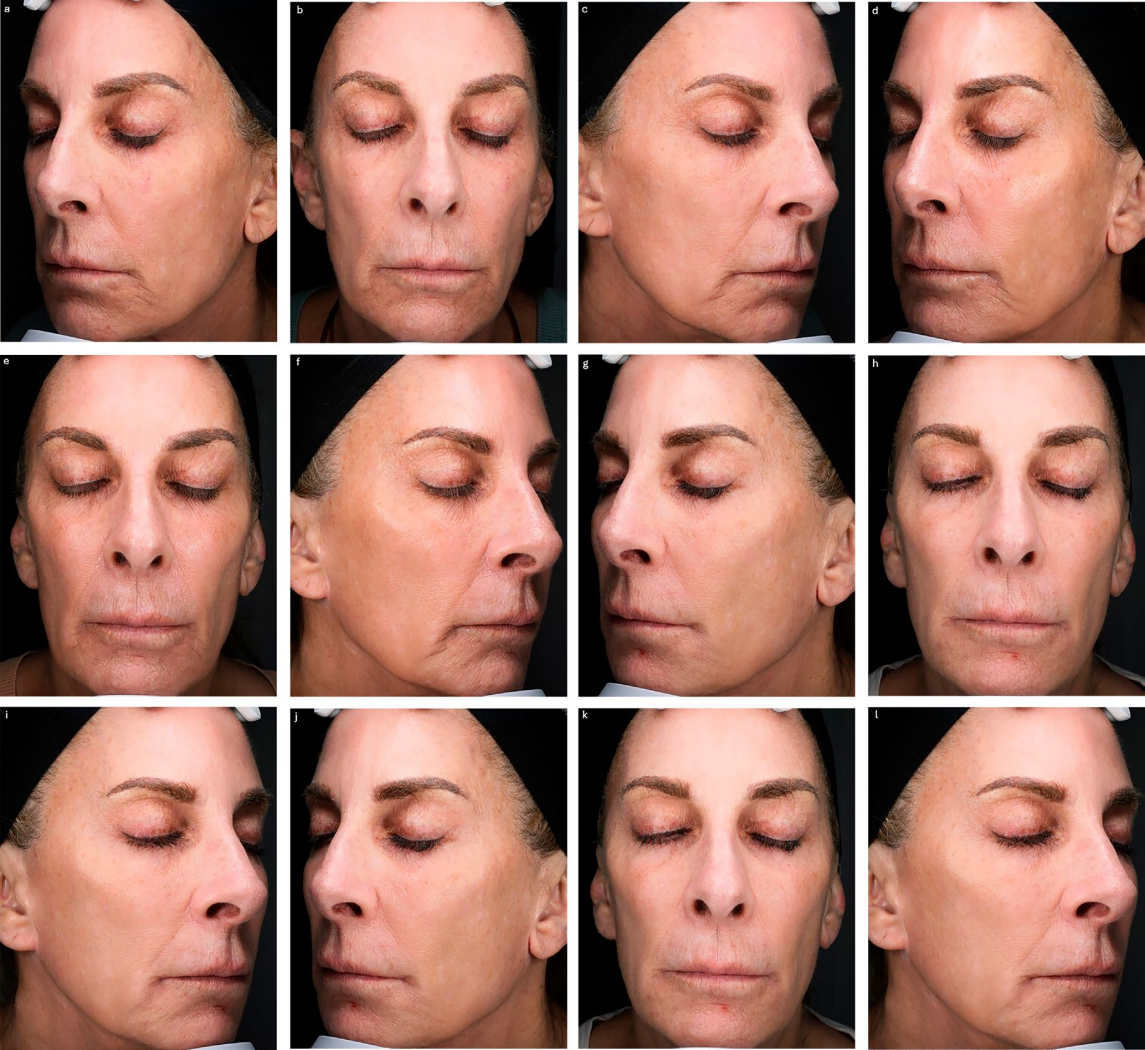
A 62‐year‐old female who lost 30lbs (13.60 kg) over a 6‐month period. At baseline/visit 1 (a–c) the patient received poly‐L‐lactic acid (PLLA‐SCA, Sculptra Aesthetic) on the right (9 mL) and left (8 mL) side of the face, along with hyaluronic acid injections (Restylane Classyc) on the right (1 mL) and left (1 mL). At visit 2 (4 weeks post‐baseline) (d–f), the patient received PLLA‐SCA on the right (9 mL) and left (9 mL), along with hyaluronic acid injections (Restylane Contour) on the right (1 mL) and left (1 mL). At visit 3 (8 weeks post‐baseline) (g–i) the patient received PLLA‐SCA on the right (9 mL) and left (9 mL). Patient at visit 4 (27 weeks post‐baseline) (j–l). Photos courtesy of Dr. Michael Somenek. The patient provided signed consent for their facial images to be published.

**FIGURE 6:**
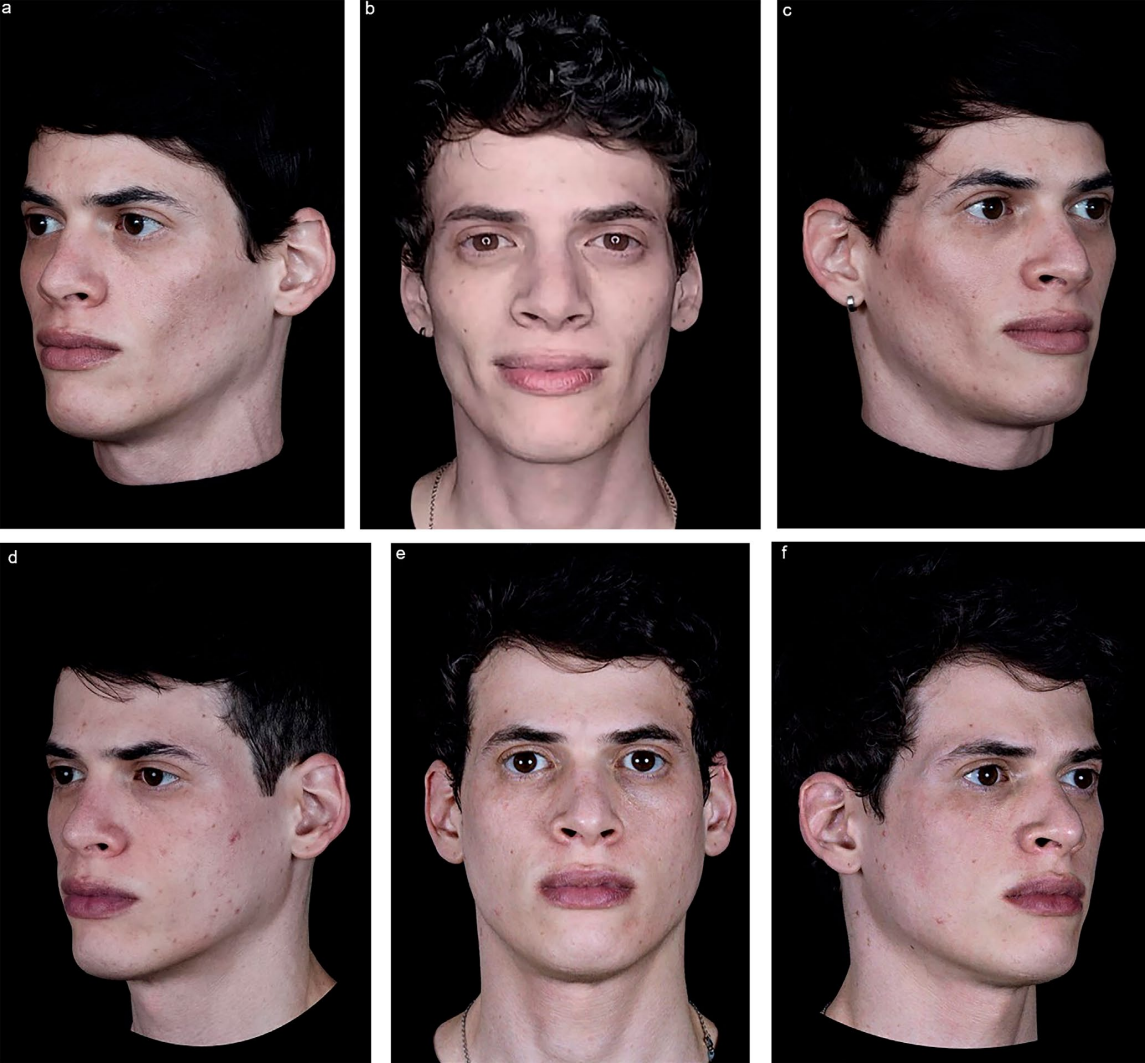
A 35‐year‐old male who lost 19.8lbs (9 kg) over 5 months. The patient received a total of 5 vials of PLLA‐SCA over a period of 45 days. Pre‐treatment (a–c). Twelve‐months post‐treatment (d–f). Photos courtesy of Dr. Luiz Avelar. The patient provided signed consent for their facial images to be published.

**FIGURE 7:**
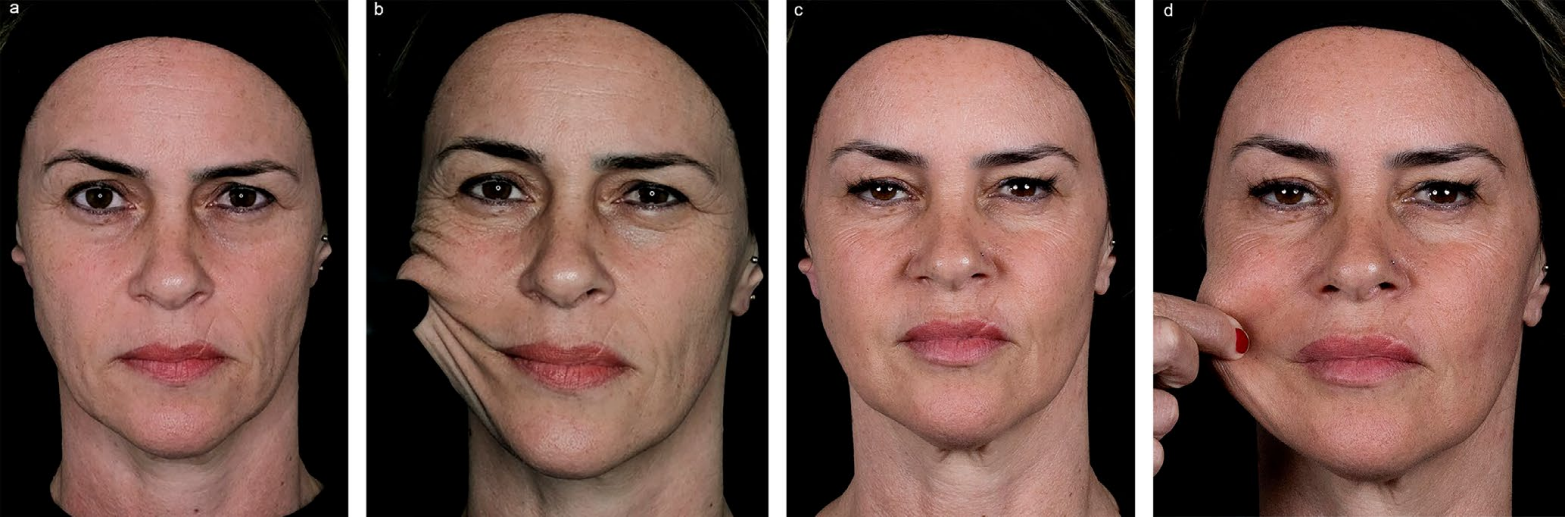
A 58‐year‐old female who lost 110.2lbs (50 kg) over 11 months. The patient received a total of 5 vials of PLLA‐SCA. Pre‐treatment (a–b). Six months post‐treatment (c–d). Photos courtesy of Dr. Luiz Avelar. The patient provided signed consent for their facial images to be published.

**FIGURE 8:**
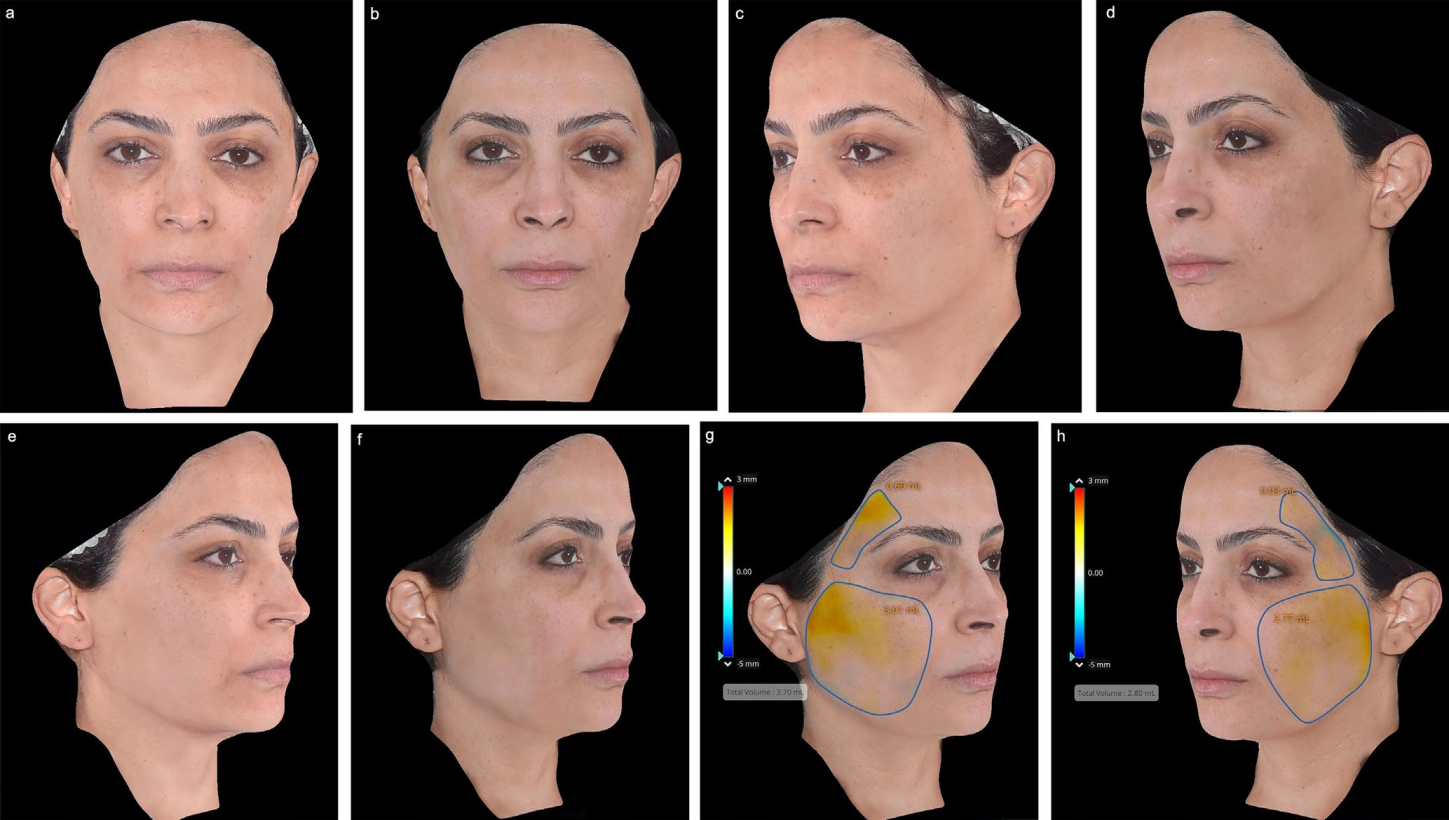
A 37‐year‐old female who lost 22lbs (10 kg) over 9 weeks. At baseline/visit 1 (a, c, e, g) the patient received 1 vial (10 cc dilution) of poly‐L‐lactic acid (PLLA‐SCA, Sculptra Aesthetic) and this regimen was repeated every 6 weeks for a total of 4 vials. As a maintenance treatment, the patient received two additional vials, once every year after the initial course for a total of 6 vials. Patient at visit 6 (b, d, f, h; 32 weeks post‐baseline). Total volume increase in the right temple and lateral cheek = 3.7 cc (g). Total volume increase in the left temple and lateral cheeks = 2.8 cc (h). Photos courtesy of Dr. Maria Angelo‐Khattar. The patient provided signed consent for their facial images to be published.

Following the meeting, panelists were asked to submit exemplary cases of mdWL patients treated in their aesthetic practices. Figures 5, 6, 7, 8 display the aesthetic journeys of mdWL patients treated with a variety of injectables (e.g., neurotoxins, fillers, biostimulators).

## 4 Discussion

Many concepts related to managing mdWL patients in aesthetics remain unexplored or are poorly reported in the literature. Relatedly, several misconceptions were identified and discussed during the group session. For example, the concept that the pathophysiology of mdWL and the subsequent tissue changes are similar to that of bariatric surgery is not always accurate. Practitioners must be aware of the complexity surrounding the treatment of patients using weight loss medications, whether they are being used primarily for health reasons (i.e., comorbidities associated with obesity) or aesthetic reasons [22]. Bariatric surgery patients typically meet higher established BMI cutoffs (e.g., > 35 kg/m^2^, or a BMI between 35 and 40 kg/m^2^ with comorbidities) and often experience a larger and more rapid weight loss that may necessitate plastic surgery to fully address aesthetic concerns (e.g., skin laxity) [23]. These patients are often inflammatory and may display poor wound healing [24]. In contrast, mdWL patients tend to have a broader range of initial BMIs; their weight loss typically occurs slower due to different metabolic pathways, and these medications are associated with systemic anti‐inflammatory actions [23]. For this reason, panelists agreed that employing a comprehensive, multi‐modal treatment approach is critical for the aesthetic management of mdWL patients.

Although research comparing signs of accelerated facial aging in patients following traditional weight‐loss methods (e.g., bariatric surgery [e.g., laparoscopic banding, Roux‐en‐Y gastric bypass, or laparoscopic sleeve gastrectomy]) versus mdWL is preliminary and ongoing, there are some important differences between these populations that may suggest they need individualized care. For example, bariatric surgery usually results in major weight loss (e.g., ≥ 50% loss in BMI) [25], which may contribute to severe skin laxity requiring surgical excision. Conversely, mdWL is usually associated with mild‐to‐moderate weight loss (i.e., ≥ 5%–20% BMI) [22], resulting in mild‐to‐moderate skin laxity. In premenopausal women and men with sufficient skin envelopes, mild‐to‐moderate cases of skin laxity may respond well to non‐surgical treatment options, such as biostimulators and/or energy‐based devices. Moreover, weight loss usually occurs more rapidly following bariatric surgery compared to mdWL, which may further increase the likelihood of observing more severe skin laxity in post‐bariatric patients [26]. Furthermore, mdWl patients may be at an increased risk of regaining weight compared to bariatric patients, as up to 50% of mdWL patients regain weight within 1 year of stopping treatment, while bariatric patients have been shown to keep the weight off for up to 10 years. If this finding is confirmed in larger, prospective trials, this may suggest that bariatric patients are more suitable for re‐volumizing procedures compared to mdWl patients. Bariatric patients may also have increased cardiovascular risk factors and obesity‐related comorbidities compared to mdWL patients, which may require specific pre‐/post‐treatment considerations to reduce the risk of adverse events and optimize results [24]. Lastly, given the significant amount of weight loss associated with bariatric patients, they will likely benefit more from pan‐facial rejuvenation and volumization as more facial areas will show deficits (midface, temporal region, periorbital, perioral, jawline, neck) compared to mdWL patients, who show volume loss primarily in the midface. The skin of bariatric patients may also display significant morphometric changes in the collagen and elastic systems, compared to mdWL patients [27].

A second misconception is that all patients undergoing prescriptive weight loss management experience accelerated facial aging. Facial aging is a multifactorial process influenced by the complex interplay of various tissue planes in the face [23, 28]. Panelists agreed that in the RWL population, the deflation of the superficial and deep fat pads of the face, the quality of the skin, as well as the sequelae of rapid weight loss possibly affecting muscles may all play a role in the appearance of accelerated facial aging. The major differential between bariatric and medication RWL groups is the quantity of weight loss, where mdWL patients typically lose 5%–20% of their initial BMI, and bariatric patients lose ≥  50% of their excess weight [29, 30]. As discussed in the consensus, patients who are losing large amounts of weight will likely experience dramatic changes to their facial appearance as the fat pads are depleted against skin that is now less contractile. However, with a significant proportion of mdWL patients experiencing smaller amounts of total weight loss and their overall weight loss journey occurring slowly, leading to a less drastic change in appearance.

A third misconception is that patients do not perceive the aesthetic effects (e.g., loss of skin radiance, reduced facial definition, early signs of facial aging) of RWL. Investigators have compared soft tissue facial changes and their correlation with actual versus apparent age among patients with massive and non‐massive weight loss. Their findings revealed that massive weight loss patients appeared 5.1 years older than their actual age, whereas non‐massive weight loss patients were 1.2 years older than their actual age following bariatric surgery [31]. In a subsequent prospective cohort study, facial age perception among morbidly obese patients was evaluated further. The mean facial age perception before versus after bariatric surgery was 40.8 versus 43.7 years. In this study, investigators observed that patients > 40 years of age were more susceptible to changes in facial volume caused by weight loss compared with younger individuals. The authors speculated that this observation can be explained by the fact that patients > 40 years of age have already encountered facial aging in addition to changes after weight loss, which may accentuate an older perceived age [32]. However, as RWL patients often have an innate fear of regaining weight, many may find the resulting physical changes to act as positive reinforcement on their weight loss journey. For some patients, looking like they lost weight may outweigh concerns about signs of accelerated facial aging (e.g., hollowing, gauntness, loose skin) [10].

A fourth misconception is that patients must be done with their weight loss journey before starting their aesthetic journey. Early intervention can help mitigate common facial aging sequelae or, at the very least, optimize facial skin health during the process [33]. From an aesthetic standpoint, patients beginning weight loss medications should be offered consultation with a dietitian to optimize their intake of essential nutrients and proteins that are critical for collagen production and skin quality. Additionally, they should be referred for resistance‐based exercises to help maintain skeletal muscle mass, which can decrease and even lead to sarcopenia in some patients. Clinicians can and likely should offer aesthetic solutions early in the weight loss process, such as optimizing topical skincare regimens, using biostimulatory treatments (e.g., poly‐L‐lactic acid injections [PLLA‐SCA, Sculptra Aesthetic]), hyaluronic acid‐based fillers, intra‐dermal hydration using micro‐droplets of hyaluronic acid (e.g., Skinboosters), and incorporating energy‐based devices (e.g., lasers, radiofrequency, microneedling). An argument brought up in the consensus panel against early intervention is that patients may not have achieved their optimal weight loss and thus their aesthetic goals will change. While this is a valid concern, since the full effect of weight loss on the face cannot be predicted, this approach can be compared to the treatment of peri‐ or post‐menopausal women, who often present with skin changes and concerns before they have undergone hormonal optimization. Furthermore, the time required for biostimulation to take place should be factored into the treatment regimen. By providing patients with structured treatment regimens throughout their weight loss journey, their skin health can be optimized, and in some cases, the effects of weight loss on facial aging may be minimized. Clinicians should use their expert judgment and clinical experience to develop a treatment plan that meets the patient's needs, goals, and expectations, whether that is before, during, or at the end of their weight loss journey.

Although patient involvement was included in the early stages of the consensus exercise (i.e., via interviews), patient and public representatives were not included as voting members, nor did they hold positions on the steering committee. This may limit the accessibility of this information for such audiences. Research has found that the involvement of patients and members of the public as partners in guideline formation and consensus processes is rarely found [14], however, future studies could improve on these models by including them as direct contributors. Choosing the number of panelists can be challenging for consensus exercises, as there is no relevant sample size calculation to conduct nor an industry gold standard [14]. The working group was limited to 10 panelists due to practical considerations and implications for the organizational process. For example, the likelihood of everyone participating equally in the decision‐making process declines as the number of panelists increases. However, larger groups enhance the range of viewpoints that can be considered when discussing complex issues [34]. Therefore, the results of this meeting may need to be extrapolated to patients and providers in different regions. In addition, given the relative recency of government approval for prescription weight loss medications, including geographical variances in drug availability, the present recommendations may require review and amendment as new information becomes available. Although none of the panelists dropped out of this exercise, it may be advisable for future studies to oversample to compensate for possible attrition each round (e.g., up to 25%).

This study represents the first global consensus‐based guidelines for understanding and managing the aesthetic needs of the mdWL patient. The key selection and critical timing of aesthetic treatments throughout the mdWL journey are presented. This guidance is supplemented with expert perspectives that are supported by up‐to‐date, peer‐reviewed data. Lastly, treatment recommendations were supported by real‐world cases. Future directives include developing a photo‐numeric scale for rating the degree and severity of facial aesthetic changes in mdWL patients and a treatment guideline/algorithm based on significant factors identified in this consensus.

## Author Contributions

**Andreas Nikolis:** conceptualization (lead), investigation, resources, supervision, writing – review and editing. **Kaitlyn M. Enright:** conceptualization, data curation, formal analysis, writing – original draft preparation. **Inna Prygova:** conceptualization, resources. **Sabrina G. Fabi:** writing – review and editing (equal). **Michael Somenek:** writing – review and editing (equal). **Hugues Cartier:** writing – review and editing (equal). **Luiz Avelar:** writing – review and editing (equal). **Johnny Franco:** writing – review and editing (equal). **Alessandra Haddad:** writing – review and editing (equal). **Maria Angelo‐Khattar:** writing – review and editing (equal). **Jeff Huang:** writing – review and editing (equal). **Tyler Safran:** writing – review and editing (equal). **Steven Dayan:** writing – review and editing (equal).

## Ethics Statement

As this manuscript describes the results of a consensus meeting, it does not constitute human subjects research and is therefore exempt from requiring independent review by a research ethics board. For all presented cases, written consent was provided, by which the patients agreed to the use and analysis of their data and publication of their full‐face photographs.

## Conflicts of Interest

Andreas Nikolis is or has been a consultant, investigator, trainer, and speaker for Galderma (Lausanne, Switzerland), Allergan (Dublin, Ireland), Prollenium (Ontario, Canada), and Merz (Frankfurt, Germany). Sabrina Fabi is or has been a speaker, investigator, and consultant for Galderma and Merz. Luiz Avelar is or has been a speaker, investigator, and consultant for Galderma. Michael Somenek is or has been a speaker, investigator, trainer, and consultant for Galderma. Hugues Cartier is or has been an investigator for Galderma. Maria Angelo‐Kathar is or has been a speaker for Galderma. Johny Franco is or has been a speaker for Galderma, Jeff Huang is or has been a speaker for Galderma, Alessandra Haddad is or has been a speaker, investigator, and consultant for Galderma Steven Dayan is or has been a consultant, researcher, and speaker for Galderma. Inna Prygova is an employee of Galderma.

## Supporting information


Table S1.

**Table S2.** ACcurate COnsensus Reporting Document (ACCORD) checklist.

**Table S3.** Pre‐meeting survey.

**Table S4.** Post‐meeting survey.

## Data Availability

Reports detailing the market research and patient interviews can be obtained by contacting the respective publishers (www.miinews.com and www.MAC‐Smarter‐Research.co.uk).
