# Supplementary material for: Consensus Statements on Managing Aesthetic Needs in Prescription Medication‐Driven Weight Loss Patients: An International, Multidisciplinary Delphi Study
Source: J Cosmet Dermatol. 2025 Mar 26;24(4):e70094. doi: 10.1111/jocd.70094 (PMC11938202; doi:10.1111/jocd.70094)
Supplement: Supplementary file 1 — Table S1. [file JOCD-24-e70094-s001.docx]

**Supplemental Material 1.** Consensus group members and their respective roles.

| **N^o^** | **Member initials** | **Steering committee** | **Panelist**  **(voting member)** | **Other (specify)** |
| --- | --- | --- | --- | --- |
| 1 | A.N. |  |  | Moderator |
| 2 | K.E. |  |  | Facilitator |
| 3 | S.F |  |  |  |
| 4 | M.S. |  |  |  |
| 5 | H.C. |  |  |  |
| 6 | L.A |  |  |  |
| 7 | J.F. |  |  |  |
| 8 | A.H. |  |  |  |
| 9 | M.K. |  |  |  |
| 10 | J.H. |  |  |  |
| 11 | I.P. |  |  |  |
| 12 | S.D. |  |  |  |
